# Sophisticated Study of Time, Frequency and Statistical Analysis for Gradient-Switching-Induced Potentials during MRI

**DOI:** 10.3390/bioengineering10111282

**Published:** 2023-11-03

**Authors:** Karim Bouzrara, Odette Fokapu, Ahmed Fakhfakh, Faouzi Derbel

**Affiliations:** 1 Laboratory of Signals, Systems, Artificial Intelligence and Networks, Technopark of Sfax, Sakiet Ezzit, Sfax 3021, Tunisia; smarts.lab@crns.rnrt.tn (K.B.); contact@enetcom.usf.tn (A.F.); 2 Department of Electrical Engineering, National Engineering School of Sousse, University of Sousse, Erriadh, Sousse 4023, Tunisia; 3 UMR CNRS 7338 Biomécanique and Bioingénierie, University of Technology of Compiègne, Dr. Schweitzer Street, 60200 Compiègne, France; odette.fokapu@gmail.com; 4 Laboratory of Innovative Technologies, University of Picardie Jules Verne, UR UPJV 3899, Aisne IUT Campus Cuffies-Soissons, 13 Avenue François Mitterrand, 02880 Compiègne, France; 5 National School of Electronics and Telecommunications of Sfax, Technopole of Sfax, Sfax 3018, Tunisia; 6 Smart Diagnostic and Online Monitoring, Leipzig University of Applied Sciences, Wachterstraße 13, 04107 Leipzig, Germany

**Keywords:** induced potentials, MRI, time and frequency analysis, stationarity test, KPSS test, surrogates, biomedical engineering, image and signal processing, medical image analysis and medical decision-making

## Abstract

Magnetic resonance imaging (MRI) is a standard procedure in medical imaging, on a par with echography and tomodensitometry. In contrast to radiological procedures, no harmful radiation is produced. The constant development of magnetic resonance imaging (MRI) techniques has enabled the production of higher resolution images. The switching of magnetic field gradients for MRI imaging generates induced voltages that strongly interfere with the electrophysiological signals (EPs) collected simultaneously. When the bandwidth of the collection amplifiers is higher than 150 Hz, these induced voltages are difficult to eliminate. Understanding the behavior of these artefacts contributes to the development of new digital processing tools for better quality EPs. In this paper, we present a study of induced voltages collected in vitro using a device (350 Hz bandwidth). The experiments were conducted on a 1.5T MRI machine with two MRI sequences (fast spin echo (FSE) and cine gradient echo (CINE)) and three slice orientations. The recorded induced voltages were then segmented into extract patterns called “artefact puffs”. Two analysis series, “global” and “local”, were then performed. The study found that the temporal and frequency characteristics were specific to the sequences and orientations of the slice and that, despite the pseudo-periodic character of the artefacts, the variabilities within the same recording were significant. These evolutions were confirmed by two stationarity tests: the Kwiatkowski–Phillips–Schmidt–Shin (KPSS) and the time-frequency approach. The induced potentials, all stationary at the global scale, are no longer stationary at the local scale, which is an important issue in the design of optimal filters adapted to reduce MRI artifacts contaminating a large bandwidth, which varies between 0 and 500 Hz.

## 1. Introduction

Magnetic resonance imaging, like all other imaging techniques, is designed to support research into diseases. A particular feature of MRI is the high contrast of the smooth tissues produced. This means that even the smallest differences in the body’s soft tissues (brain, abdominal organs, spinal cord) are clearly visible. Tumors and inflammatory changes, muscles, tendons, intervertebral discs and joints can, therefore, be particularly well-represented. Magnetic resonance imaging (MRI) techniques, like other medical approaches, such as dental implantology [1], the vascularization of engineered tissues [2] and synthetic bone graft substitution (BGS) [3], are constantly evolving with the aim of improving the image quality and broadening the spectrum of applications. These developments have led to considerable improvements in the spatial and temporal resolution of diagnostic MRI images, and to the development of functional and interventional MRIs. Unfortunately, these advances generate sources of artefacts that “pollute” the electrophysiological signals (EPs) acquired simultaneously [4,5,6]. Indeed, better spatial and temporal resolution requires increasingly strong gradients with shorter and shorter rise times, sometimes associated with higher magnetic fields (3-7T). EPs are essential for patient monitoring and image synchronization in all types of MRI examinations [7,8]. They are also used as a source of additional information in functional MRI. EPs “pollution” is mainly due to the interaction between the electromagnetic devices needed to construct the MRI images and the EPs acquisition devices used for patient monitoring [9,10]. The signal s(t) collected by the electrodes can be modeled as a linear combination of the desired signal EPs, and three main sources of noise [4]: $s\left(t\right)=s_{EPs}\left(t\right)+n_{RF}\left(t\right)+n_{MHD}\left(t\right)+n_{GA}\left(t\right)$. $s_{EPs}\left(t\right)$ is the electrophysiological signal, $n_{RF}\left(t\right)$ represents the tensions induced by the RF pulses, $n_{M}HD\left(t\right)$ represents the tensions induced by the RF pulses and $n_{GA}\left(t\right)$ are the voltages induced by the gradient switches. The $n_{RF}\left(t\right)$ and $n_{M}HD\left(t\right)$ components can be properly reduced by filtering. The voltages induced by gradient switching are difficult to remove because they have large amplitudes and frequencies that fall within the bandwidth of the electrophysiological signals. For several decades the electrocardiogram (ECG) [11] has been used during MRI examinations for patient monitoring and image acquisition synchronization. However, this technique is performed using electronic devices with a low bandwidth (1 Hz–60 Hz) which does not allow provision of a good diagnostic quality ECG. Indeed, a diagnostic ECG requires a wide bandwidth (0.05–150 Hz). Recent studies show the interest in and difficulties associated with this type of collection [12,13,14,15]. The surface electromyography signal is another interesting example in the functional MRI of neurological pathologies. Combining the information from the image of a muscle section and the EMG signal collected simultaneously on the surface of the same muscle could provide diagnostic assistance. At present, obtaining a clean EMG signal in the presence of gradients is a major difficulty; its amplitude is low (few µv) and its bandwidth is (0.05–500 Hz). Research in this field confirms the interest in and difficulties of this type of investigation [16,17,18]. In the cited works, EMG was used only as a control for muscle activity. The authors did not perform a quantitative analysis to correlate the EMG and fMRI signals, due to gradient-induced potentials affecting the EMG signals. The variations in the time-frequency characteristics of the EMG in the presence of gradient artefacts are even more complex. To eliminate the induced potentials that strongly contaminate the broadband electrophysiological signals, it would be interesting to understand the behavior of these artefacts systematically generated by gradient switching. The results of this type of investigation could contribute to the development of new analogue and digital processing tools leading to better quality EPs. The study we propose here falls within this framework and focuses on the characterization of the temporal and frequency variations of the $n_{GA}\left(t\right)$ component. The novelty of this work lies in its focus on addressing a specific problem related to the contamination of electrophysiological signals (EPs) during magnetic resonance imaging (MRI) procedures. In this paper, we present a study of induced voltages collected in vitro. In the laboratory, we developed a device that allows collection of only the potentials induced by gradient switching according to different imaging protocols. After analysis of the temporal and frequency properties, we formulated a study of the stationarity of these induced voltages. The fundamental notion that informs the modeling of a temporal process is that of stationarity. Two approaches for testing stationarity were applied to the rewired artefacts: the KPSS stationarity test and the time-frequency stationarity test. The first part of the paper briefly presents the theoretical basis of the two stationarity test methods used. The second part of the paper describes the process of collecting the induced potentials, the method of analysing their variabilities in the time and frequency domains, which is widely used in several fields, like the monitoring and early warning of mine rockbursts [19], and the algorithm for the stationarity study. The results and a discussion of the observations are presented in the last section.

## 2. State of the Art of Stationaity Test

The stationarity of electrophysiological signals such as EMGs, EEGs and ECGs have often been studied in order to better develop tools for extracting relevant information [20,21,22]. The stationarity of these same signals recorded in MRI does not seem to have been studied, probably due to the complexity of the different noise sources involved. It seemed appropriate to first study the noise generated by gradient switching using an in vitro approach.

### 2.1. The Kwiatkowski–Phillips–Schmidt–Shin Stationarity Test

By definition, a signal is considered strictly stationary if, and only if, its statistical moments are independent of time. In practice, it is virtually impossible to verify stationarity in the strict (or strong) sense, mainly because a real physical signal can never be stationary in the strict sense. For this reason, it makes sense to define stationarity in the weak, i.e., second-order, sense. In practice, an acquired signal can be assimilated to a time series whose “trajectory” we observe and analyze in order to qualify it as stationary if it is likely to result from a stationary process. According to the definition of stationarity in the weak sense, non-stationarity can arise from a time dependence of the first-order moment (the expectation) and/or a time dependence of the variance or the auto-covariance. Kwiatkowski et al. proposed hypothesis tests to verify under the null hypothesis that a series is stationary in level $\eta_{\mu }$ or around a trend $\eta_{\tau }$ [23]. To this end, a time series is modeled as follows:

(1) $$y_{t}=\delta t+\xi_{t}+\varepsilon_{t},$$

where $\varepsilon_{t}$ is a stationary error, δt is a deterministic trend and $\xi_{t}$ is a random walk given by the following equation:

(2) $$\xi_{t}=\xi_{t-1}+\mu_{t}\;$$

where $\mu_{t}$ iid (0,$\sigma_{\mu }^{2}$): under the null hypothesis, the signal is trend-stationary, i.e., $\sigma_{\mu }^{2}$ = 0. In the special case where δ = 0, the KPSS test can be used to check that the signal is weak-sense stationary.

### 2.2. Stationarity Test with a Time-Frequency Approach

The time-frequency approach for testing the stationarity of a time series recommended by Jun et al. [24,25] is very briefly presented below. The starting point of this approach is that second-order stationary processes are a special case of the class of harmonizable processes where time-varying spectra can be defined. When the process under analysis is stationary, its time-varying spectra can be reduced to the classical power spectral density (PSD). This is true for a good choice, such as for the Wigner–Ville Spectrum (WVS). The basic idea underlying the approach used here is, therefore, that, considered over a given period of time, a process is said to be stationary with respect to this scale of observation if its time-varying spectrum does not support any evolution—in other words, if the spectra at all different times are statistically similar to the global spectrum obtained by marginalization. This idea is not new, but the approach advocated is based on the meaning of the difference “local vs. global”.

#### 2.2.1. The Time-Frequency Approach

The first element required for the test is a time-frequency representation susceptible to guarantee robust subsequent processing. The choice here will be made on a multi-window spectrogram, which has the advantage of being a good estimator of the theoretical Wigner–Ville spectrum [26]. Given a signal x(t), the spectrogram is estimated according to [27]:

(3) $$S_{x,K}\left(t,f\right)=\frac{1}{k}\sum_{k=1}^{K}S_{x}^{\left(h_{k}\right)}\left(t,f\right)$$

where $\sum_{k=1}^{K}S_{x}^{\left(h_{k}\right)}$ represents the K spectrograms computed on the signal x(t), taking as short-term windows the successive terms $h_{k}\left(t\right)$ of an (orthonormal) basis of Hermite functions:

(4) $$S_{x}^{\left(h_{k}\right)}\left(t,f\right)={\left|\int x\left(s\right)h_{k}\left(s-t\right)e^{-i2\pi fs}ds\right|}^{2}$$

In practice, the average (4) refers to a reduced number of windows, usually between 5 and 10. Another essential element of any window analysis is, of course, the size of the windows, irrespective of their shape. In the present context, the possibility to vary this size provides an intrinsic degree of freedom to the method in order to adjust the horizon of the local analysis with respect to the global time scale set by the total observation time.

#### 2.2.2. Surrogates

The idea of the test is to identify the concept of stationarity with the equivalence of global and local spectral properties. In order to have a quantifiable basis for comparison between the global and local characteristics, the proposed approach is to associate the observed signal with a “stationary” reference in order to be able to reject the stationarity hypothesis, if necessary. To this end, the authors use the interpretation that, for the same spectrum mean, a non-stationary signal differs from a stationary counterpart by a temporal structure whose signature is found in the spectral phase. Thus, given a single observed signal x(t), it is possible to associate a battery of substitutes [28,29] $s_{j}\left(t\right);\;j=1,\;\ldots ,\;J$, each having the same power spectrum as the original signal but a stationary time content. It would be enough to replace the original phase of the spectrum by a random phase.

#### 2.2.3. Distances

The idea is to compare the local spectra with the global spectrum. For this purpose, we defined the quantities marginalized in time as follows:

(5) $${\langle S_{y,k}\left(t_{n,f}\right)\rangle }_{n}=\frac{1}{N}\sum_{n=1}^{N}S_{y,k}\left(t_{n,f}\right)$$

Since the signal y(t)=x(t) for J substitutes $y\left(t\right)=s_{j}\left(t\right);\;1,\;\ldots ,\;J)$, the different time-frequency spectrum was only evaluated at N times $t_{n}$, which are a fraction of the equivalent width of the short-term windows. The “distances” J + 1 between the local and the global spectra are derived from this equation:

(6) $$\left\{C_{n}^{\left(y\right)}=D\left(S_{y,K}\left(t_{n},.\right),{\langle S_{y,K}\left(t_{n},.\right)\rangle }_{n}\right),n=1,\ldots ,N\right\}$$

where D (.,.) stands for some dissimilarity measure (or “distance”) in frequency.

In order to choose a measure of dissimilarity between spectra, the authors adopt a pragmatic attitude which consists in considering the simplest “distances” that have already proved their efficiency in similar contexts. A good choice of measurement is based on two spectra G (f) and H (f),

(7) $$k\left(G,H\right)=k_{KL}\left(\tilde{G},\tilde{H}\right)\cdot \left(1+k_{LSD}\left(G,H\right)\right)$$

Combining the Kullback–Leibler divergence

(8) $$k_{KL}\left(\tilde{G},\tilde{H}\right)=\int_{\Omega }\left(\tilde{G(f)}-\tilde{H(f)}\right)log\left(\frac{\tilde{G}\left(f\right)}{\tilde{H}\left(f\right)}\right)df$$

Applied to the normalized spectrum $\tilde{G(f)}$ and $\tilde{H(f)}$ from G(f) and H(f) and the log-spectral deviation

(9) $$k_{LSD}\left(G,H\right)=\int_{\Omega }\left|\log\frac{G(f)}{H(f)}\right|df$$

#### 2.2.4. Stationarity Test

Let us consider $s_{j}\left(t\right),j=1,\ldots ,J$ as the J substitution signals obtained as just described. When they are analyzed as explained above for the original signal x(t), we finally end up with a new collection of distances depending on both the time indices and the randomizations.

(10) $$\left\{c_{n}^{s_{(}j)}=k\left(S_{s_{j},K}\left(t_{n},.\right),{\langle S_{s_{j},K}\left(t_{n},.\right)\rangle }_{n}\right),n=1,\ldots ,J\right\}$$

To measure the fluctuations in time of the divergences $c_{n_{n}}^{\left(.\right)}$ between local and global spectra, one can use the distance $l_{2}-$ defined by Equation (16):

(11) $$L\left(g,h\right)=\frac{1}{N}\sum_{n=1}^{N}{\left(g_{n}-h_{n}\right)}^{2}$$

For each pair of sequences $\left\{\left(g_{n}h_{n}\right);n=1,\ldots ,N\right\}$. Regarding the intrinsic variability in the proxy data, the dispersion of the divergences under the null hypothesis of stationarity can be measured by the distribution of the J empirical variances

(12) $$\left\{\theta_{0}\left(j\right)=L\left(c^{\left(s_{j}\right)},{\langle c^{\left(s_{j}\right)}\rangle }_{n=1,...N}\right),j=1,...J\right\}$$

The distribution is used to determine the threshold γ over which the null hypothesis is rejected. The effective test is, therefore, based on the statistics

(13) $$\theta_{1}=L\left(c^{\left(x\right)},{\langle c^{\left(s_{j}\right)}\rangle }_{n=1,\ldots ,N}\right),j=1,\ldots ,J$$

And takes the form of the unilateral test:

(14) $$\theta_{1}>\gamma :“non-stationarity^{”};\;\theta_{1}<\gamma :“stationarity^{”}$$

#### 2.2.5. Index of Non-Stationarity

Test (20) is used to determine the non-stationarity of a signal in terms of its achievement. In the non-stationary case (where the null hypothesis is rejected), it is then possible to define a non-stationary index (INS) according to the following relation:

(15) $$INS=\sqrt{\frac{\theta_{1}}{\frac{1}{J}\sum_{j=1}^{J}\theta_{0}\left(j\right)}}$$

## 3. Signal Acquisition and Treatment

### 3.1. Recording of Induced Potentials

**Figure 1:**
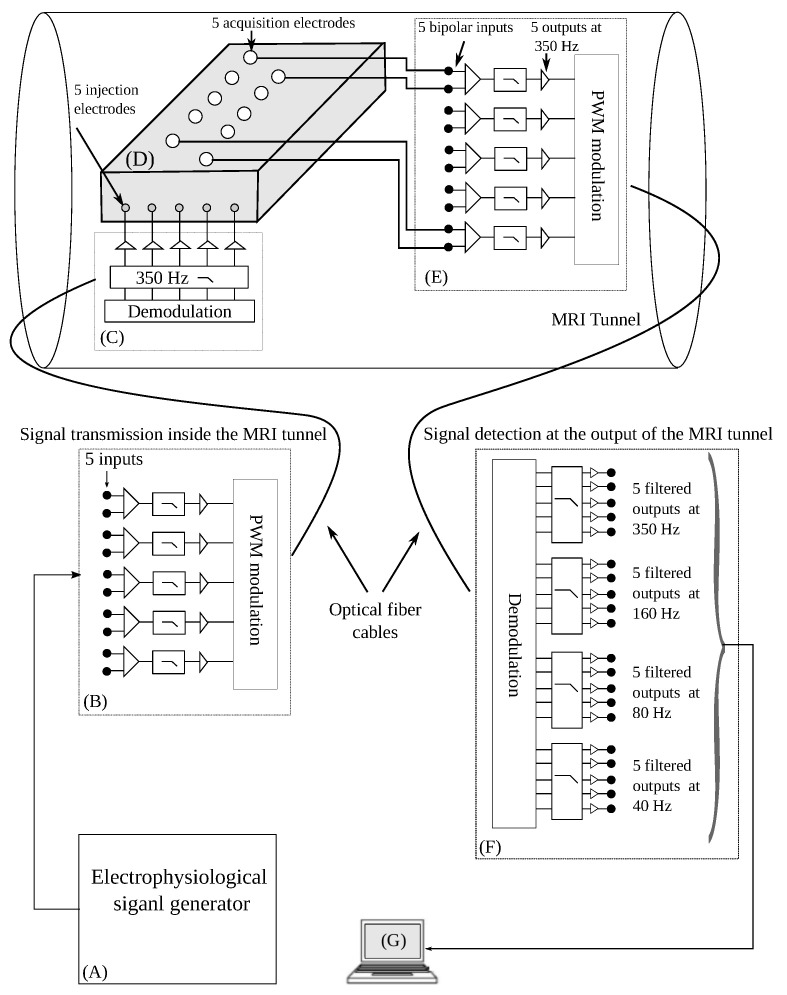
Experimental bench with two “transmitter-receiver” modules: (**B**,**C**) for signal transmission, and (**E**,**F**) for detection. From (**A**), five signals can be injected simultaneously into the tunnel via the transmitter (**B**), the optical fiber and the receiver (**C**). These contaminated signals at the conductive fabric (**D**) are detected by the second transmitter (**E**) and transmitted to the outside of the tunnel via a second optical fiber and the second receiver (**F**). When the generator (**A**) is off and the MRI sequences are activated, the system collects the potentials induced by the gradient switches. The contaminated signals or induced potentials are stored and processed by the station (**G**).

An experimental bench was built in our laboratory to collect the in vitro potentials induced by gradient switching (Figure 1). A detailed description of this bench was published in a previous paper [30]. From an electrophysiological signal generator (A), five signals can be injected simultaneously into the tunnel via the transmitter (B), the optical fiber and the receiver (C). These signals contaminated at the level of the conductive tissue (D), which is placed at the center of the magnet, are detected by the second transmitter (E) and transmitted to the outside of the tunnel via a second optical fiber and the second receiver (F). The data is stored and processed by station G. The non-MRI-compatible elements (A), (B), (E) and (G) are placed outside the MRI chamber.

The bandwidth of the set extends from 0.05 Hz to 350 Hz. The bench has 20 channels divided into four frequency bands (40 Hz, 80 Hz, 160 Hz and 350 Hz). It is, thus, possible to analyze the changes in the signal parameters according to the sequences, but also within the different frequency bands. This bench offers different types of experiments. It consists of two MRI-compatible “transmitter-receiver” modules. The first allows EPS signals with known characteristics to be introduced into the MRI tunnel. The signals are injected into a sample of conductive tissue placed in the tunnel. The second module allows the signals to be collected after they have been contaminated by artefacts generated by the imaging sequences owing to the electrodes placed on the conductive tissue. When no signal is injected into the tunnel, the potentials induced after activation of the MRI sequences can be collected. This type of experiment was used in the present work. The study focused on the induced potentials collected at the output of the 350 Hz (broadband) filter, which, therefore, contains a maximum of noise generated by the gradients. The experiments were conducted on a 1.5T MRI system (GE Signa HDxt 1.5T, GE Healthcare) equipped with a 33 mT/m gradient system. To simulate a human body, a conductive tissue model was placed in the MRI tunnel. It was made from salt, gelatine powder and water. By varying the concentrations of salt in the gel, different conductivities of the medium were obtained. For this study, the conductivity was 348 Ω·cm. The induced potentials were acquired with three carbon electrodes (3MTM RedDotTM radiolucent electrode). The induced potentials were sampled at 5 kHz and recorded for a duration of 10 s. The MRI sequences used were FSE and CINE in three slice orientations. Fast spin echo (FSE) (Fov = 30 × 30 cm, TR/TE = 500/12 ms, Matrix = 448 × 512) and cine gradient echo (CINE GE) (Fov = 34 × 25 cm, TR/TE = 9.4/5.1 ms, Matrix = 256 × 128) were used as the MRI sequences.

### 3.2. Pre-Treatment

**Figure 2:**
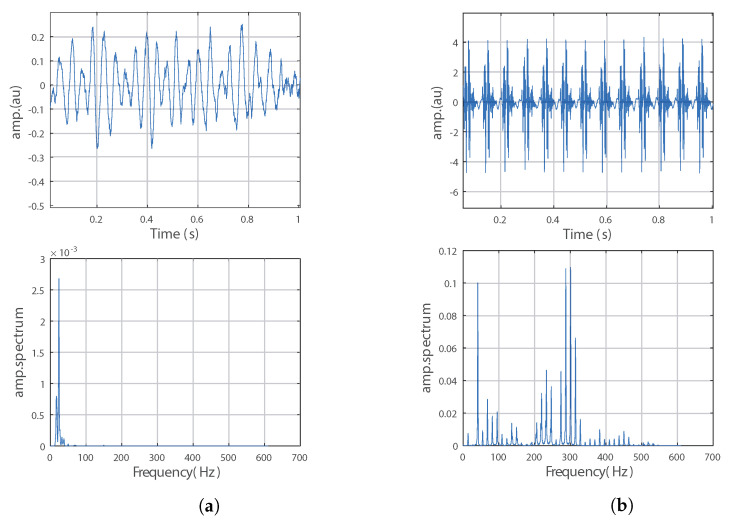
(**a**) Non-sequenced noise and its frequential representation, and (**b**) induced potentials FSE.

Figure 2a shows an example of low-frequency noise recorded without MRI sequence activation. Figure 2b illustrates an example of an induced potential where the amplitude modulation by the noise seen in Figure 2a is observed. There are also bursts of artefacts that appear periodically. These two features were exploited in the studies presented below. The studies focused on a 5-s recording duration for global analysis, and on the artefact puffs for local analysis.

A recording without sequence activation was also performed in order to observe the disturbances caused by the static field B0 and the rest of the measurement environment.

#### 3.2.1. Normalization

In order to process the potentials collected in different sequences and slice orientations, the normalization of the data is essential in order to transform the amplitude values of the induced potentials from their original values into comparative scales. In this study the Z-score normalization used was set by the following formula:

(16) $$Y\left(s\right)=\frac{X(s)-\mu }{\sigma }$$

Y(s) represents the normalized induced potential, X(s) is the original induced potential μ and σ represents the mean and standard deviation.

#### 3.2.2. Puff Extraction

**Figure 3:**
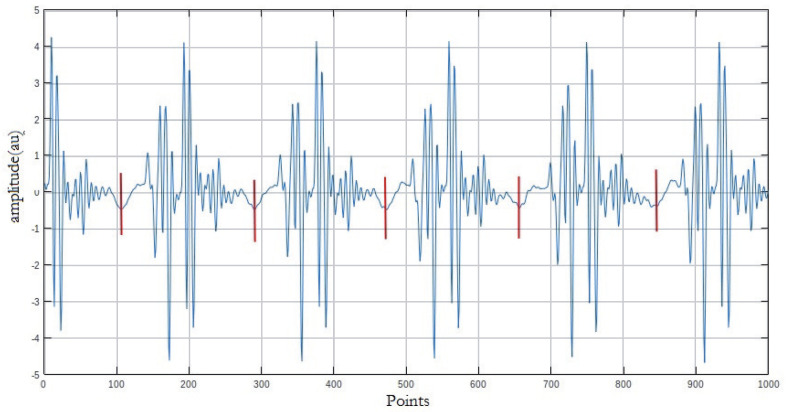
Signal segmentation principle.

In order to precisely delineate the artefact bursts, we performed a manual segmentation, which is more accurate for delineating the puffs (Figure 3).

The operation was applied to 5 s of induced potential recorded as indicated above for each of the two sequences and according to the three orientations. This gave a total of three “noise signals” per sequence, i.e., six recordings to be segmented. In all, 80 puffs were extracted from each of the six segments, i.e., a total of 480 puffs to be analyzed.

#### 3.2.3. Time and Frequency Analysis of Induced Potentials

The aim here was to verify the expected properties and to highlight the inter-sequence and intra-sequence variabilities. The estimated time and frequency domain characteristics are given below:

- RMS values of the global signal and RMS values of the different bursts.
- Estimation of the average curve of the chirps, and calculation of its RMS value.
- Measurement of the similarity between the puffs by calculating the mean square error between each puff and the mean curve according to the following equation:

(17) $$ern=\frac{1}{\bar{y}}\sqrt{\sum_{1}^{N}{\left(\hat{y_{i}}-y_{i}\right)}^{2}}$$

This value is normalized to the range $\tilde{y}=y_{max}-y_{min}$.

- The calculation of the power spectral density (PSD) is performed by the Welche–WOSA method, and estimation of the characteristic parameters, the average frequency, the maximum amplitude frequency and the standard deviation of the overall spectrum and on the set of puffs are obtained by segmentation.

### 3.3. Stationarity Study

#### 3.3.1. Kpss Method

The theoretical approach outlined in Section 2.1 was applied to the different 5 s recordings, which was enough to have sufficient puffs to analyse:

(a) the KPSS of the six 5 s recordings (FSE/axial/coronal/sagittal-CINE/axial/coronal/sagittal).
(b) the KPSS of the 480 extracted puffs and evaluation of the variabilities by estimating the mean values and standard deviation of the obtained series of values. The values were also grouped and graphed to highlight the degree of stationarity or non-stationarity of the different studied segments of the induced potentials.

#### 3.3.2. Surrogate-Based Method

The algorithm proposed by Bor et al. [24], which is based on the briefly presented approach in Section 2.2, was adapted and applied to our records. The program is implemented in the Matlab language and contains different functions. Below is a presentation of the main steps of the algorithm:

(1) Time-frequency representation: The choice was made for the multi-window Wigner–Ville spectrogram, having successive short-term windows of a Hermite function base. This allows the possibility to adapt the window sizes of our recordings to the MRI sequences.
(2) Surrogate generation: A set of surrogates each having the same power spectral density as the original signal was created. This was achieved by keeping the Fourier transform modulus unchanged but replacing its phase with another randomly taken on [−π,π].
(3) The stationarity test is based on the distances between the local and global spectra. The distance calculation was carried out by combining the Kullback–Leiber divergence (KL) and log spectral deviation (LSD) methods.

The studied induced potentials show an amplitude modulation by a pseudo sinusoidal low-frequency noise (Figure 2a). Since our induced potentials are similar to the examples of signals used by the authors of the surrogate approach to validate their approach, we were inspired by their approach for the choice of the parameters for the stationarity study. These parameters are as follows:

- Number of substitutes: 5000;
- Number of windows: 5;
- Window size range: [0.03:0.04:0.005:0.07:0.075] adjusted for slice orientation.

The possibility of varying this size provides a degree of freedom intrinsic to the method to allow the local analysis to be adjusted in relation to the global time scale set by the total observation time.

## 4. Results and Discussion

**Figure 4:**
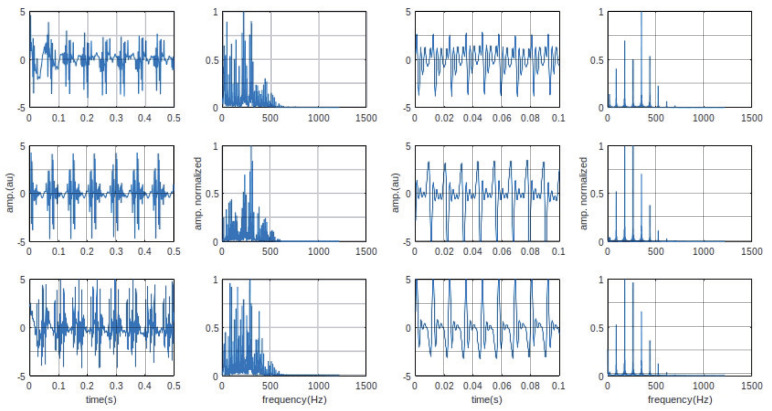
Recorded induced potentials: representation in time and frequency domains. Columns 1 and 2, potentials induced by the FSE sequence (coronal/axial/sagital). Columns 3 and 4, potentials induced by the CINE sequence (coronal/axial/sagittal).

The temporal and frequency analysis and stationarity studies were applied to a series of induced potential recordings to highlight the variability of the characteristics of the gradient-induced potentials. The different graphs allow a qualitative observation of these variabilities. Table 1, Table 2, Table 3 and Table 4 summarize the main parameters calculated for a quantitative analysis of the observed variabilities. The six recordings, according to two sequences and three slice orientations processed throughout this work, are shown in Figure 4. It can be observed that the pseudo-periodicity of each of the six recordings was confirmed by a spectrum of amplitude lines more or less rich according to the imaging sequence. The frequencies of the amplitude maxima are different according to the slice orientations.

### 4.1. Puff Analysis

**Figure 5:**
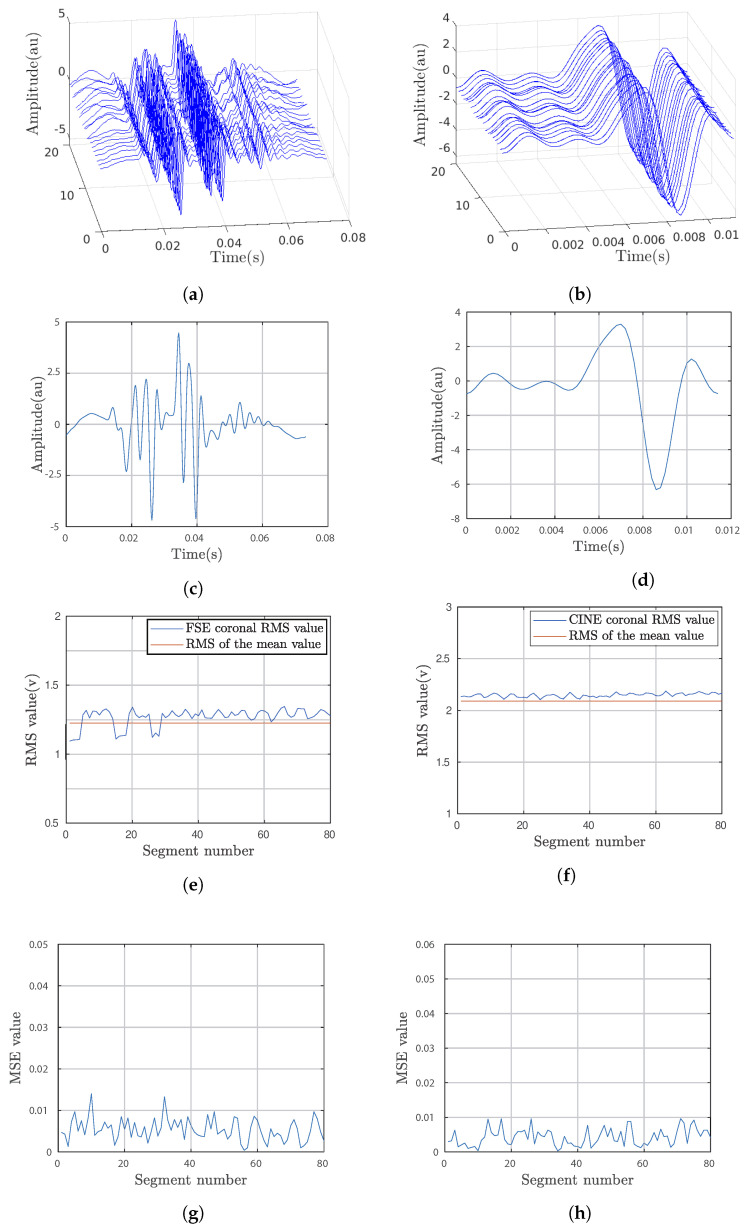
Puffs extracted from the induced potential recordings after activation of the coronal orientation for the FSE and CINE sequences. (**a**,**b**) are the 3D representation of FSE and Cine sequences respectively, (**c**,**d**) are the average puffs of FSE and Cine, (**e**,**f**) the variability in the RMS values around the RMS value of the mean puff and (**g**,**h**) is the variability in the average od quadratic deviation of each puff.

The puffs of the potentials obtained by segmentation on the previously described six recordings are shown in Figure 5. The first row shows a 3D representation of the extracted puffs (20 as an example) for each of the FSE and CINE sequences, respectively. The second row shows the respective average puffs. The third row shows the variability in the RMS values of the 80 puffs around the RMS value of the mean puff. The fourth row shows the variability in the average quadratic deviation of each puff from the average puff curve.

The average puffs were estimated for the coronal orientation of the FSE and CINE sequences. The analysis of these puffs shows the variability of the features within a sequence. The RMS values calculated for each puff were compared to the RMS value of the average puff curve. We also compared the root mean square deviation between each puff and the mean puff evaluated from the set of puffs.

The qualitative analysis of the different graphs in Figure 5 shows that the waveforms of the average puffs from one orientation to another are very different, which is an expected result. The puffs appear in a regular way, but their shape is not identical, as shown in the MSE curves. Table 1 summarizes the values of the calculated RMS and MSE parameters.

The RMS values calculated over the entire duration of the recordings indicate that the orientation of the slices does not determine the level of noise power; it is higher for the axial FSE than the CINE. The dispersion of the RMS values of the puffs is greater for the coronal FSE orientation (0.0644) but the mean value 1.2684 is close to the RMS value calculated on the global recording (1.2566). The variability degree in the morphology of the puffs within the same sequence is more or less significant depending on the sequence and the cutting orientation. The RMS and MSE curves show significant variability for the coronal orientation of the FSE sequence despite the periodic character of the puffs.

For all six recordings, we noted that the estimated average frequency parameters varied significantly from one puff to another for the same slice orientation. This indicates that an in-depth study of the stationarity of the induced potentials is an avenue to explore. The average values of the frequency parameters were calculated in order to compare them with the global values; differences of around 5% were noted.

### 4.2. Global and Local Power Spectral Density

**Figure 6:**
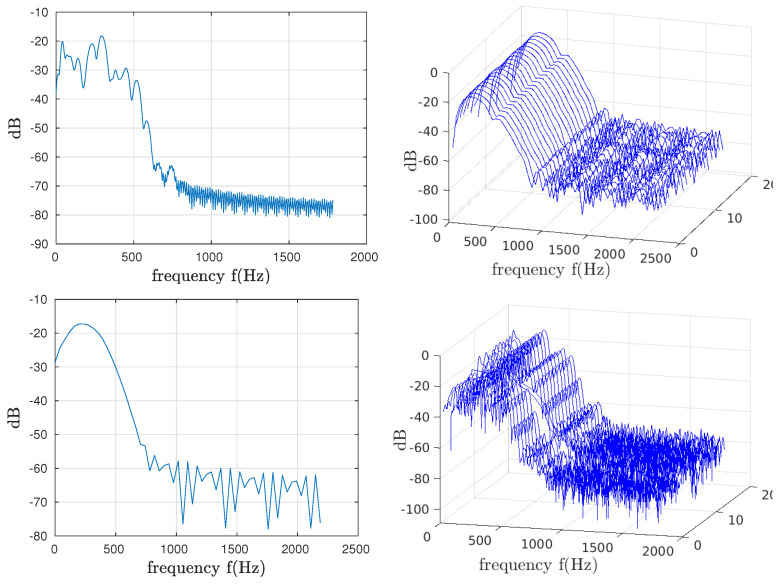
Spectral analysis of the coronal orientation for FSE and CINE sequences. Column 1: global spectrum for each FSE and CINE, respectively. Column 2: spectrum of local segments.

The calculated power spectral densities of the induced potentials are shown in Figure 6. They are calculated over the total duration of the recordings (global PSD) and for each series of puffs (local PSD). The first interesting observation on the global PSDs we can make is the very significant variability in the frequency parameters within the same sequence. For example, for the FSE sequence, the average frequency, fmean, is 234.97 Hz, 145.82, and 114.26 for the three orientations, coronal, axial, and sagittal, respectively.

Table 2 shows that the puff-by-puff calculated parameters (local analysis) occupy a quite large interval for each frequency range (min–max). The frequency of the maximum amplitude varies significantly from one puff to the next, whatever the sequence and orientation. This parameter is influenced by the low frequency modulation mentioned in the beginning of the paper. This parameter, whose variations do not depend solely on the morphology of the induced potential, must be analyzed with caution.

### 4.3. Stationarity

The stationarity study was motivated by the frequency parameters variability observed on the local power spectral densities. The results of this investigation are displayed in this subsection. Two stationarity test processes were proposed: a KPSS test and a time-frequency test. The study was carried out both globally (the total duration of the recording was considered) and locally (each puff extracted from the same recording was analyzed). The local study allows observing the evolution of the stationarity criteria. It was interesting to compare two stationarity-testing methods, one of which uses frequency properties.

For the global study, both methods lead to the results displayed in Table 3, showing the stationarity of the induced potentials whatever the sequence and the cutting orientation. For the KPSS test, the statistical value is effectively below the threshold of 0.1460. The recordings made according to two sequences and three slice orientations, allow highlighting the intra- and inter-sequence variability. The local analysis shows significant levels of variability in the features that cannot be identified in the global analysis. This point was confirmed by both the KPSS and the time-frequency stationarity tests.

For the time-frequency test, a large number of substitutes was taken (JJ = 5000). The stationarity was verified for the six records, and the values of theta and INS were, in fact, lower than the threshold values estimated by the algorithm.

For the local analysis (puff-by-puff evolutionary observations), the stationarity tests results are shown in Table 4. Some puffs are not stationary. It is to be noted that all puffs tested stationary by the time-frequency method were confirmed by the KPSS statistical test. The reverse is not true; in fact, 97.5% of puffs tested stationary by the KPSS method were not confirmed by the time-frequency method. For a given record, the variation range of the stationarity thresholds are generated automatically, while the mean values and the values of the estimated dispersion are shown in Table 4. Depending on the orientation of the cut, the variability in the test parameters can be significant. For example, for the axial orientation, we have a range of [min–max] = [0.1851–0.8744], with a dispersion of 0.18 for the KPSS test; this was confirmed by the threshold values obtained by the time-frequency method. For this slice orientation, no puffs were tested stationary. In contrast, for the coronal orientation, both methods indicate stationarity for 78 of the 80 puffs analyzed.

It should be noted that only the recordings obtained with the FSE sequence are shown because, for the CINE sequence, the time-frequency method did not provide usable results, the duration of the potentials being very short for this type of approach.

**Figure 7:**
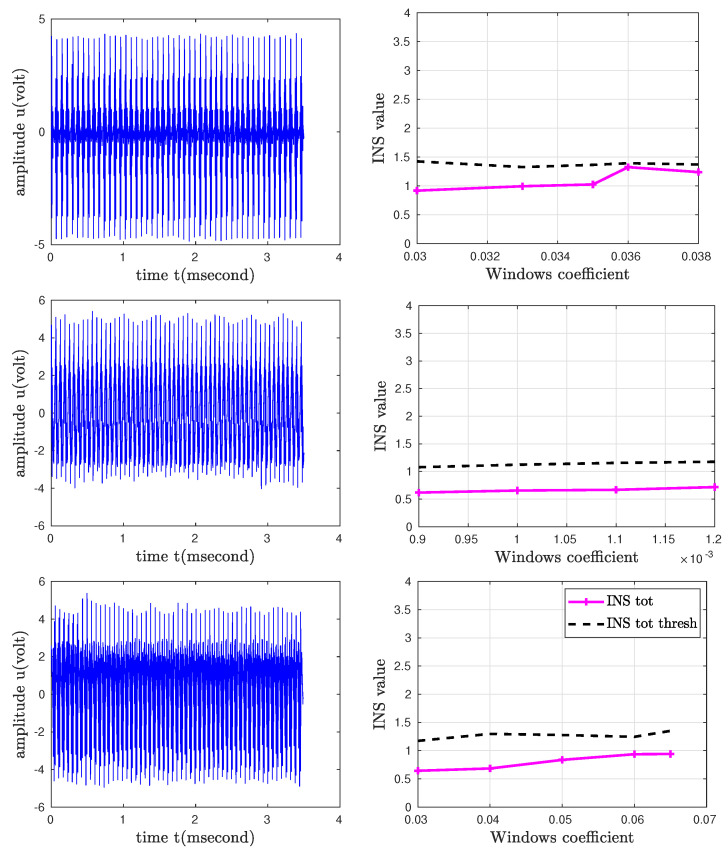
Graphical illustration of INS values in relation to the threshold for the FSE sequence orientations.

For the time-frequency test, the window size is an important parameter in the evaluation of stationarity. The choice of the windows applied was guided by the nature of our “noise signals”, which are amplitude modulated, similar to those tested by the authors of this approach [24]. Knowing that for the global study, all our “noise signals” are stationary by the time-frequency approach, we analyzed the influence of the window size for each of the three slice orientations. The evolution of the INS is a function of the number of windows and their sizes. The results are shown in Figure 7.

It was noted that, for the range of variation [0.003–0.05], the obtained curves remain below the INS threshold and show that the induced potentials remain stationary. This approach, which takes into account the frequency characteristics of the signals, is interesting, but the choice of the number of windows and their size is a delicate issue. The number of substitutes is also a parameter that could influence the stationarity results. We tested with 50 and 5000 surrogates and verified the null hypothesis of stationarity. To evaluate the null hypothesis of stationarity, we have taken up the idea advocated in [25], i.e., the representation of the asymptotic histograms of the distributions of θ relative to the surrogates and its fitting by the gamma distribution.

**Figure 8:**
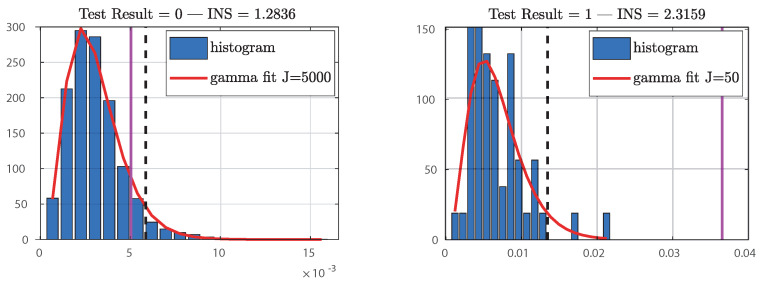
Histogram of Θ(j), surrogate-based distribution and its gamma fitting.

An illustration is given in Figure 8 for two induced potential puffs—one is stationary but the other is not. For the example shown in Figure 8 (5000 and 50 surrogates), the magenta plot is in the middle of the distribution, which proves that the null hypothesis of stationarity is met. The last example shown in Figure 8 is a case of non-stationarity; the statistical value in magenta is far from the distribution.

## 5. Conclusions

These signals are useful to monitor the subject and are also used in the MRI examination process itself, for example, as a source for triggering observation sequences, and in its interpretation by correlation with information obtained in functional MRI. Unfortunately, the technical constraints specific to MRI give rise to sources of artefacts which ’pollute’ the electrophysiological signals collected simultaneously. A knowledge of the variability in the characteristics of the artefacts that cause signal disturbances is essential in the choice of strategies to adopt for the development of signal cleaning algorithms. The novelty of this work lies in its systematic investigation of the contamination of EPs by gradient-induced artefacts during MRI scans. It involves the development of a specialized device, detailed analysis of the temporal and frequency properties, and the application of stationarity tests. In this work, an analysis of the induced potentials generated by the gradient switches collected during MRI examination was investigated. The temporal, frequency and statistical characteristics of these artefacts were determined globally and locally. The global analysis was performed on the total recording time and the local analysis on segments extracted from the same recording. The segments designated as puffs of the induced potentials were isolated in accordance with the temporal pseudo-periodicity that characterize these artefacts. It should be noted that the study presented in this paper is just a first step, as the induced studied potentials, collected in vitro, do not have all the characteristics of induced potentials collected in vivo. Forthcoming studies will first focus on the collection of induced potentials in vivo, then on digital filters’ modeling. An experimental protocol to isolate segments of the induced potentials generated during the collection of the electrophysiological signals will be developed. The characterizations of the induced potentials is specific to such sequences as MRI sequences. Other MRI sequences, particularly those that generate more artefacts, like true FISP or EP sequences, will be tested in a future study.

## Figures and Tables

**Table 1:** Illustration of RMS and MSE values of FSE and CINE sequences.

		FSE			CINE		
		**Axial**	**Coronal**	**Sagital**	**Axial**	**Coronal**	**Sagital**
**RMS**	Global	1.9022	1.2566	1.7004	1.4715	2.2606	2.1468
	[min–max]	[0.8770–1.0017]	[1.0947–1.3463]	[1.2018–1.3360]	[1.3902–1.4610]	[2.1046–2.1855]	[2.0844–2.1394]
	Mean–stdev	0.9393–0.0279	1.2684–0.0644	1.2770–0.0293	1.4154–0.0152	2.1463–0.0181	2.1028–0.0125
**MSE**	[min–max]	[0.0001–0.0133]	[0.0004–0.0140]	[0.0003–0.0109]	[0.0003–0.0151]	[0.0003–0.0096]	[0.0004–0.0099]
	Mean–stdev	0.0029–0.0036	0.0054–0.0027	0.0036–0.0031	0.0053–0.0037	0.0043–0.0025	0.0043–0.0027

**Table 2:** Representation of the mean and maximum frequencies and standard deviation for the FSE and CINE puffs.

	FSE			CINE		
**Frequency**	**Coronal**	**Axial**	**Sagital**	**Coronal**	**Axial**	**Sagital**
$F_{mean}$	[246.163–254.390]	[87.66–97.451]	[121.364–161.663]	[217.309–231.005]	[254.220–295.720]	[230.406–237.216]
$F_{max}$	[280.681–287.959]	[9.765–9.765]	[9.548–10.184]	[234.375–234.375]	[234.375–234.375]	[234.375–234.375]
stdev	[0.0013–0.0013]	[0.004–0.005]	[0.0033–0.0049]	[0.0043–0.0051]	[0.0013–0.0015]	[0.0079–0.0084]

**Table 3:** Results of the stationarity tests for the two sequences FSE and CINE with the KPSS method and the time-frequency method.

		FSE			CINE		
**Stationarity Test**		**Coronal**	**Axial**	**Sagital**	**Coronal**	**Axial**	**Sagital**
KPSS test	Statistical value	0.0678	0.0921	0.0403	0.0098	0.0019	0.0020
Surrogates	Theta	0.0089	0.0048	0.0033	0.0072	0.0032	0.0055
	Threshold	0.2698	0.0994	0.4134	0.0311	0.0573	0.0320
	INS	0.0776	0.0941	0.1208	0.0779	0.0377	0.0652
	INS threshold	1.3457	1.3314	1.3341	1.6109	1.5754	1.5632

**Table 4:** Test results for the FSE puffs.

		KPSS Test	Surrogate Test			
**FSE**		**Statistical Value**	**Theta**	**Threshold**	**INS**	**INS Threshold**
Coronal	Mean–stdev	0.1280–0.1472	0.0051–0.0009	0.0058–0.005	1.2607–0.0984	1.5920–0.0413
Axial	Mean–stdev	0.4376–0.1838	0.0041–0.0004	0.0067–0.0011	1.3963–0.4480	0.8594–0.3205
Sagittal	Mean–stdev	0.3142–0.1475	0.0117–0.0011	0.0067–0.0011	1.9565–0.5376	1.2336–0.2032

## Data Availability

The original contributions presented in this study are included in this article. Further inquiries can be directed to the corresponding authors.
